# Evaluation of the fully automated urine particle analyzer UF‐1500

**DOI:** 10.1002/jcla.24993

**Published:** 2023-12-02

**Authors:** Yoshifumi Morita, Rin Yokoyama, Masami Tanaka, Naru Nakatsuka, Takashi Hisasue, Yoshikazu Ono, Makoto Kurano

**Affiliations:** ^1^ Department of Clinical Laboratory The University of Tokyo Hospital Tokyo Japan; ^2^ Department of Clinical Laboratory Medicine Graduate School of Medicine, The University of Tokyo Tokyo Japan

**Keywords:** UF‐1500, urine, urine particle analyzer, urine sediment

## Abstract

**Background and Aims:**

This study primarily assessed the performance of the UF‐1500, the novel and compact model of the fully automated urine particle analyzer and evaluated its performance against the existing UF‐5000 instrument.

**Materials and Methods:**

A total of 648 residual urine specimens were randomly collected and examined using both the UF‐1500 and UF‐5000 instruments as well as manual microscopy. For each parameter, the concordance rates and detection accuracy of the UF‐1500 against manual microscopy were compared with the UF‐5000.

**Results:**

The concordance rates between the UF‐1500 and manual microscopy were 75.3%–98.5%. The UF‐1500 concordance rates within one group agreement were observed to be >90%, for all parameters except for YLCs. The differences within one group agreement between the UF‐1500 and manual microscopy were insignificant, in comparison to the UF‐5000, with exceptions noted for ECs and YLCs. The sensitivity and specificity of the UF‐1500 for RBCs, WBCs, Squa.ECs, and BACT exceeded 80%, while the positive predictive values of ECs and CASTs were below 70%.

**Conclusion:**

The UF‐1500 exhibited a performance that was comparable to the existing instrument, the UF‐5000, and was suitable to be introduced in clinical practice. For the samples with suspected false‐positive or false‐negative results, a manual microscopic examination is required for accurate testing.

## Graphical abstract

The novel and compact model of the fully automated urine particle analyzer UF‐1500 was evaluated. The performance of the UF‐1500 is comparable to that of the existing UF‐5000 analyzer. The UF‐1500 was suitable to be introduced in clinical practice, but a manual microscopic examination is necessary for the samples with false‐positive or false‐negative results.

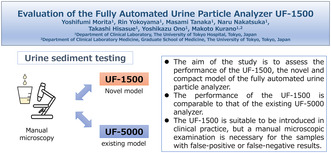

## 1 INTRODUCTION

Urine sediment analysis is a clinically significant and common practice used to screen for urinary and renal diseases. It is traditionally carried out through manual microscopy^1^ and is known to be time‐consuming and labor‐intensive. Several instruments based on either imaging systems or flow cytometry have been developed to automate urine sediment tests, in order to address the limitations associated with manual microscopy.^2^, ^3^ Using these instruments could reduce the number of manual microscopy reviews, thereby improving workflow efficiency.^4^ Furthermore, the clinical application of automated analyzers can enable the standardization of urinary sediment detection and counting. However, the performance of these new instruments should be validated against manual microscopy for clinical application, since manual microscopic examination is still considered the gold standard for urine sediment tests.

The UF‐5000 (Sysmex Corporation) is an established instrument based on flow cytometry analysis. Several clinical studies have demonstrated its applicability in diagnosing urinary tract infections,^5^, ^6^, ^7^, ^8^ glomerular hematuria,^9^, ^10^ and also for identifying atypical/malignant cells.^11^, ^12^, ^13^ However, due to its large footprint, the UF‐5000 might not be an ideal choice for some laboratories.

As a result, the UF‐1500 (Sysmex Corporation), a novel compact model of the fully automated urine particle analyzer, was developed to meet the needs of smaller labs and preserve the efficacy of the conventional instrument. The purpose of this study was to evaluate the performance of the compact UF‐1500 analyzer.

## 2 MATERIALS AND METHODS

### 2.1 Samples

A total of 648 residual urine samples were randomly collected from outpatients and inpatients at the University of Tokyo Hospital. After mixing the samples, 10 mL of urine was aliquoted into a tube for analysis. All urine samples were promptly tested using the UF‐1500, the UF‐5000, and manual microscopic examination.

The current study was performed under the ethical guidelines outlined in the Declaration of Helsinki. Informed consent was obtained in the form of an opt‐out option on a website as follows: Patients were informed about the study on the website, and those who did not wish to be enrolled in the study were excluded. The University of Tokyo Medical Research Center Ethics Committee (2019300NI‐6) approved the study design.

### 2.2 UF‐1500 and UF‐5000 analysis

The specificity of the UF‐1500 is depicted in Table 1. The measurement and analysis principles of the UF‐1500 are consistent with the UF‐5000, thereby enabling the measurement of all the same parameters. The UF‐1500 is based on a flow cytometry method, employing a blue semiconductor laser. The type and quantity of urine sediment components can be detected using forward scattered light, side scattered light, side fluorescence, and depolarization side scattered light.

**TABLE 1:** Specification comparison between UF‐1500 and UF‐5000.

	UF‐1500	UF‐5000
Size	630 (W) × 626 (D) × 630 (H) mm	760 (W) × 754 (D) × 855 (H) mm
Quantitative parameters	RBC, WBC, EC, Squa.EC, CAST, BACT	
Semi‐quantitative or qualitative parameters	WBC clumps, Non SEC, Hy.CAST, Path.CAST, X'TAL, YLC, SPERM, MUCUS	
Research parameters	RBC‐P70Fsc, RBC‐Fsc‐DW, Large RBC, Small RBC, NL RBC, Lysed RBC, Tran.EC, RTEC, SRC, Atyp.C, Cond., Osmo., DEBRIS, SF_TC, CW_TC, CB_TC, SF_OTHERS, CW_OTHERS	RBC‐P70Fsc, RBC‐Fsc‐DW, Large RBC, Small RBC, NL RBC, Lysed RBC, Tran.EC, RTEC, SRC, Atyp.C, Cond., Osmo., DEBRIS
Research information	RBC‐Info., UTI‐Info., BACT‐Info.	
Body fluid mode	No	Yes
Max processing speed (sample number / hour)	60	105
Sampling volume	0.68 mL (sampler mode) 0.52 mL (STAT mode)	0.45 mL (sampler mode, STAT mode)
Required sample volume	2.0 mL (sampler mode), 0.6 mL (STAT mode)	2.0 mL (sampler mode), 0.6 mL (STAT mode)

Abbreviations: Atyp.C, atypical cells; BACT, bacteria; BACT‐Info., Bacteria Gram dye‐affinity Information; CB_TC, CRch (BACT) total count; Cond., conductivity; CW_OTHERS, CRch (WBC) others; CW_TC, CRch (WBC) total count; EC, epithelial cells; Hy.CAST, hyaline casts; NL RBC, non lysed RBC; Non SEC, non‐squamous epithelial cells; Osmo., osmolality; Path.CAST, non‐hyaline casts; RBC, Red blood cells; RBC‐Info., RBC morphological Information; RTEC, renal tubular epithelial cells; SF_OTHERS, SFch others; SF_TC, SFch total count; SPERM, spermatozoa; Squa.EC, squamous epithelial cells; SRC, small round cells; Tran.EC, transitional epithelial cells; UTI‐Info., UTI Information; WBC, white blood cells; X'TAL, crystals; YLC, yeast‐like cells.

### 2.3 Manual microscopic examination

Manual microscopic examination was performed in accordance with the protocol outlined by the Japanese Committee for Clinical Laboratory Standards (JCCLS). After centrifuging 10 mL of urine sample for 5 min at 500*g*, the supernatant solution was decanted. The remaining 0.2 mL of urine sediment was thoroughly mixed, 15 μL was transferred onto the glass slide, and then covered by a cover glass measuring 18 × 18 mm. Blood cells (red blood cells [RBC], white blood cells [WBC]), epithelial cells (EC), (squamous epithelial cells [SEC], urothelial cells, and renal tubular epithelial cells [RTE]) were counted under a high‐power field of view (/HPF). Urinary casts were classified into one of the following types: hyaline casts, granular casts, epithelial casts, fatty casts, red blood cell (RBC) casts, white blood cell (WBC) casts, vacuolated denatured casts, crystal casts, fibulin casts, broad casts, and/or waxy casts. The total number of casts and oval fat bodies (OFB) were counted per whole field (/WF), and repeated per low‐power field (/LPF) if the number of casts exceeded 100 counts /WF. For semiquantitative grading for bacteria (BACT), crystals (X'TAL), yeast like cells (YLC), spermatozoa (SPERM) by manual microscopy, we employed the following criteria:

1+: Presence observed in each field.
2+: Multiple or scattered in clusters.
3+: Numerous.

Four or more WBCs counted by manual microscopy were reported out as “WBC clumps” by the analyzer. The total number of urothelial cells and RTEs counted by manual microscopy were reported out by the analyzer as nonsquamous EC (“Non‐SEC”). The following elements seen by manual microscopy were counted, totaled and deemed as nonhyaline cast (“Path. CAST”) by the anlayzer: granular casts, epithelial casts, fatty casts, RBC casts, WBC casts, vacuolated denatured casts, crystal casts, fibulin casts, broad casts, and waxy casts.

### 2.4 Statistical analysis

The formulae particles (p) /HPF = 0.18* p/μL and p/LPF = 2.9* p/μL were used for data conversion, enabling comparison of the results from the UF‐1500 or the UF‐5000 with manual microscopy. Concordance rates and those within one group agreement were calculated by creating box correlation diagrams to visualize the correlations between the UF‐1500 and manual microscopy. Positive criteria were defined as RBC ≥ 5 p/HPF, WBC ≥ 5 p/HPF, EC ≥ 1 p/HPF, Squa.EC ≥ 1 p/HPF, Non‐SEC ≥ 1 p/HPF, CAST ≥ 1 p/LPF, Hy. CAST ≥ 1 p/LPF, and Path.CAST ≥ 1 p/LPF.

The statistical software “EZR” (Easy R) was used to analyze the data.^14^ For each parameter, McNemar's test was used to examine the differences in concordance rates between the UF‐1500 and manual microscopy, as well as the UF‐5000 and manual microscopy. The data were considered to be statistically significant for *p* values < 0.05 in all the analyses.

## 3 RESULTS

### 3.1 Comparison of the results of quantitative parameters between the UF‐1500 and manual microscopy

**FIGURE 1:**
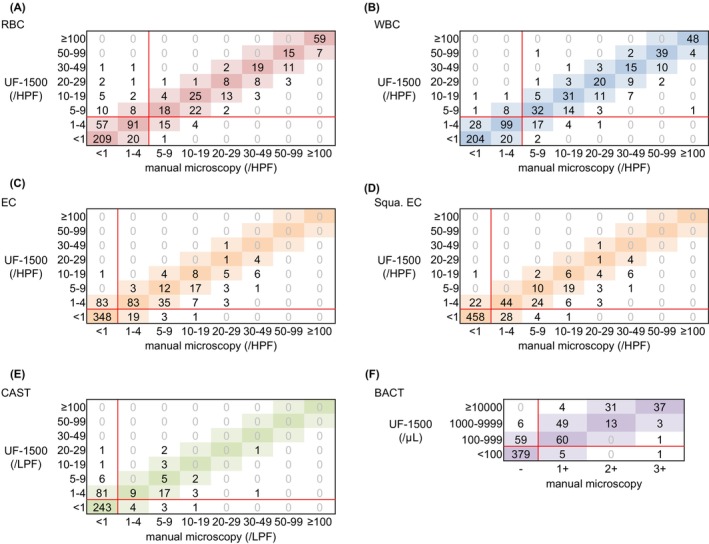
Comparison results of quantitative parameters using UF‐1500. Consistency between the UF‐1500 and manual microscopy with regards to RBCs (A), WBCs (B), ECs (C), Squa.ECs (D), CAST (E), and BACT (F) are shown in the box correlation diagrams. The red line represents the cutoff values.

Figure 1 depicts the box correlation diagrams representing the comparison results between the UF‐1500 and manual microscopy for quantitative parameters including RBCs, WBCs, ECs, squamous epithelial cells (Squa.EC), CAST, and bacteria (BACT). The concordance rates and those within one group agreement were as follows: 68.5% and 94.4% for RBCs, 75.3% and 96.0% for WBCs, 69.8% and 96.1% for ECs, 80.1% and 96.1% for Squa.ECs, 67.1% and 95.3% for CAST, and 75.5% and 98.1% for BACT, respectively (Table 2). Figure S1 depicts the box correlation diagrams between the UF‐5000 and manual microscopy. There were no significant concordance rate differences between the UF‐1500 and manual microscopy, or between the UF‐5000 and manual microscopy with regards to all quantitative parameters except for the concordance rates within one group agreement for ECs. The sensitivity and specificity for RBCs and WBCs were >90% for both the UF‐1500 as well as the UF‐5000, with a positive likelihood ratio (PLR) of >10, and the negative likelihood ratio (NLR) of <0.1 (Table 3). Meanwhile, for CASTs, the positive predictive value (PPV) and PLR on both the UF‐1500 and the UF‐5000 were as low as 32.6% and 38.0%, 3.145 and 3.987, respectively. The PPV and PLR were greater for Squa.EC compared to EC. Thus, the test performance for all parameters on the UF‐1500 and the UF‐5000 showed little difference when compared to manual microscopy.

**TABLE 2:** Comparison of the concordance rates between UF‐1500 and UF‐5000 with manual microscopy.

	Concordance rates			Concordance rates within one group agreement		
	UF‐1500 versus manual microscopy	UF‐5000 versus manual microscopy	*p* Value	UF‐1500 versus manual microscopy	UF‐5000 versus manual microscopy	*p* Value
Quantitative parameters						
RBC	68.5%	70.1%	0.194	94.4%	93.7%	0.332
WBC	75.3%	74.8%	0.787	96.0%	96.8%	0.131
EC	69.8%	67.9%	0.201	96.1%	97.8%	<0.01
Squa.EC	80.1%	81.3%	0.243	96.1%	96.6%	0.45
CAST	67.1%	71.3%	0.106	95.3%	95.3%	1.000
BACT	75.5%	75.8%	0.831	98.1%	98.6%	0.371
Semi‐quantitative or qualitative parameters						
WBC clumps	86.7%	85.0%	<0.01	N/A	N/A	–
Non SEC	83.5%	78.7%	<0.01	98.9%	99.2%	0.724
Hy.CAST	70.7%	76.9%	<0.01	95.9%	96.9%	0.386
Path.CAST	92.2%	89.1%	<0.01	98.7%	98.0%	0.134
X'TAL	90.3%	92.9%	<0.01	97.8%	98.0%	1.000
YLC	77.3%	84.0%	<0.01	85.8%	89.8%	<0.01
SPERM	98.5%	98.5%	1.000	100.0%	100.0%	N/A
MUCUS	80.2%	78.9%	0.407	N/A	N/A	–

*Note*: McNemer's test are performed to compare the concordance rates and those within one group agreement between UF‐1500 and UF‐5000 with manual microscopy.

**TABLE 3:** Performance comparison between tests with UF‐1500 and UF‐5000 against those with manual microscopy.

	RBC		WBC		EC	
	UF‐1500 versus manual microscopy	UF‐5000 versus manual microscopy	UF‐1500 versus manual microscopy	UF‐5000 versus manual microscopy	UF‐1500 versus manual microscopy	UF‐5000 versus manual microscopy
Sensitivity	91.7%	92.5%	91.6%	93.0%	89.4%	91.7%
Specificity	92.6%	90.9%	97.0%	97.2%	80.6%	75.0%
PPV	88.0%	85.8%	96.0%	96.4%	69.7%	64.7%
NPV	95.0%	95.4%	93.6%	94.6%	93.8%	94.7%
PLR	12.392	10.165	30.533	33.214	4.608	3.668
NLR	0.090	0.083	0.087	0.072	0.132	0.111

	Squa.EC		CAST		BACT	
	UF‐1500 versus manual microscopy	UF‐5000 versus manual microscopy	UF‐1500 versus manual microscopy	UF‐5000 versus manual microscopy	UF‐1500 versus manual microscopy	UF‐5000 versus manual microscopy
Sensitivity	80.2%	82.0%	84.3%	96.1%	97.1%	97.5%
Specificity	95.2%	94.2%	73.2%	75.9%	85.4%	84.5%
PPV	85.4%	83.0%	32.6%	38.0%	75.3%	74.3%
NPV	93.3%	93.8%	96.8%	99.2%	98.4%	98.7%
PLR	16.708	14.138	3.146	3.988	6.651	6.290
NLR	0.208	0.191	0.214	0.051	0.034	0.030

Abbreviations: NLR, negative likelihood ratio; NPV, negative predictive value; PLR, positive likelihood ratio; PPV, Positive predictive value.

### 3.2 Comparison results of semiquantitative or qualitative parameters between the UF‐1500 and manual microscopy

**FIGURE 2:**
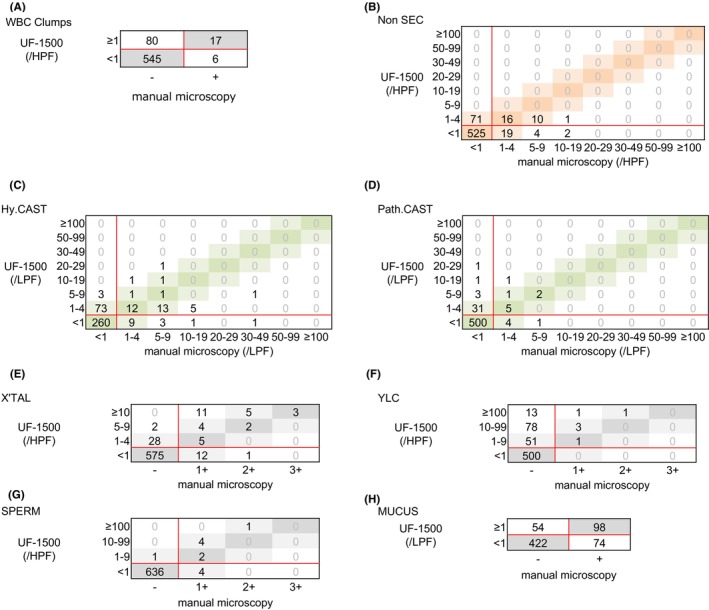
Comparison results of semiquantitative or qualitative parameters in UF‐1500. Consistency between the UF‐1500 and manual microscopy with regards to WBC clumps (A), Non‐SEC (B), Hy. CAST (C), Path. CAST (D), X'TAL (E), YLC (F), SPERM (G), and MUCUS (H) are shown in the box correlation diagrams. The red line represents the cutoff values.

Box correlation diagrams representing result comparisons of semiquantitative or qualitative parameters such as WBC clumps, Non‐SEC, Hy. CAST, Path. CAST, X'TAL, YLC, SPERM, and MUCUS, between the UF‐1500 and manual microscopy, and the UF‐5000 and manual microscopy have been depicted in Figure 2 and Figure S2, respectively. The concordance rates and those within one group agreement for the UF‐1500 and manual microscopy were 86.7% and not available (N/A) for WBC clumps, 83.5% and 98.9% for Non‐SEC, 70.7% and 95.9% for Hy.CAST, 92.2% and 98.7% for Path.CAST, 90.3% and 97.8% for X'TAL, 77.3% and 85.8% for YLC, 98.5% and 100.0% for SPERM, and 80.2% and N/A for MUCUS, respectively (Table 2). For WBC clumps, Non‐SECs, Hy.CASTs, Path.CASTs, and X'TALs, concordance rate differences between the UF‐1500 and manual microscopy and the UF‐5000 and manual microscopy were noted to be statistically significant. However, there was no significant difference in concordance rates within one group agreement for the method comparisons. For YLCs, both the concordance rate and within one group agreement were observed to be significantly different.

### 3.3 Cause analysis of specimens with discrepancies between the UF‐1500 and manual microscopic examination

Finally, we investigated potential causes for urinary component discrepancy counts between the UF‐1500 and manual microscopy. The discrepancies were defined in terms of the following criteria: positive/negative disagreement in box correlation and discordance with two or more ranks (Figures 1 and 2). The false‐positive rates and false‐negative rates (the numbers in brackets indicate the number of samples analyzed) for the UF‐1500 were as follows: 3.4% (22) and 0.8% (5) for RBCs; 0.5% (3) and 1.1% (7) for WBCs; 0.2% (1) and 0.6% (4) for ECs; 1.2% (8) and 0.6% (4) for CASTs; 0.9% (6) and 0.2% (1) for BACT; 0.3% (2) and 0.2% (1) for X'TAL: 14.0% (91) and 0% for YLCs. Among the false‐positive samples for RBCs and YLCs, an abnormal RBC/YLC fractionation error was observed in 14 of 22 samples and 21 of 91 samples, respectively.

## 4 DISCUSSION

In the present study, we evaluated the performance of the UF‐1500, the recently developed compact model of the fully automated urine particle analyzer. Despite a slightly slower processing speed compared to the UF‐5000, the new compact model may solve the installation space problem that exists with some smaller labs. The concordance rates within one group agreement between the UF‐1500 and manual microscopy were >90% except for with YLCs, the concordance rate differences were insignificant between the UF‐1500 and the UF‐5000 when compared to manual microscopy (Figures 1, 2, and Table 2). Although the PPVs of ECs and CASTs were <70%, the UF‐1500 detection accuracy for RBCs, WBCs, Squa.ECs, and BACT were sufficient and supportive of using this analyzer in clinical practice (Table 3).

The UF‐1500 exhibited high performance in counting RBCs, WBCs, and BACT. However, manual microscopic examination is recommended to count and classify RBCs with other urinary particles, such as YLCs, especially in cases when the abnormal RBC/YLC fractionation error is observed. However, concordance rates for the UF‐1500 against manual microscopy were not significantly different compared to those of the UF‐5000. In addition, although the UF‐1500 demonstrated the significant difference in the concordance rates specific to WBC clumps in the McNemar's test, which may be attributed to the presence of very small values in the cells of the contingency table, even when there was almost no difference in concordance rates. From a practical perspective, it is reasonable to consider that this difference might be not so significant.

Among the ECs, a higher concordance rate was observed for Squa.EC between the UF‐1500 and manual microscopy, compared to ECs. Moreover, the UF‐1500 demonstrated an improved concordance to manual microscopy, when compared to the UF‐5000, regarding the classification of non‐SECs, which involves the identification of urothelial cells and/or RTEs. However, both the UF‐1500 and the UF‐5000 counted lower numbers of non‐SECs compared to manual microscopy (Figure 2B). These results suggest that while automated classification and counting of squamous epithelial cells using the UF‐1500 yields reliable results, the detection accuracy for urothelial cells and RTEs remains insufficient and requires manual microscopic examination.

The performance of UF‐1500 with urinary casts was not substantial, necessitating the use of manual microscopy for reliable results. The higher number of false‐positives observed for Path.CAST compared to Hy.CAST is a contributing factor for this finding. With the UF‐1500, urinary particles trapped in mucus were thought to be incorrectly identified as Path.CASTs. With regards to the other urinary particles, the concordance rates of UF‐1500 for YLCs were lower than rates observed with the UF‐5000. False‐positive samples showing pyuria and bacteriuria suggested that bacteria or collapsed WBCs were misidentified as YLCs. Despite the near identical measurement principles of both analyzers, algorithm improvement is necessary to achieve accurate testing outcomes, given the lower concordance rate between the UF‐1500 and manual microscopy compared to the UF‐5000 and manual microscopy.

Presently, domestic laboratories follow two principal workflows for urine sediment testing combined with urine sediment analyzer and manual microscopic examination: The first measures all samples using an analyzer after the measurement with a dipstick analyzer and perform the manual microscopic examination based on a pre‐established criteria derived from the results of both the urine dipstick test and the urine sediment analyzer. Meanwhile, the second sets specific criteria based on the results of the urine dipstick test or patient information and only samples meeting these criteria are measured using the urine sediment analyzer, while other samples undergo direct manual microscopic examination. In any case, it is not feasible to report all samples requiring urinary sediment examination based only on the urine sediment analyzer measurement results. The best workflow method would combine both the automated analyzer and conventional manual microscopic examination. Urine sediment analyzers have unique characteristics that are essential for the accurate urine sediment identification. Therefore, it is critical to understand each analyzer's characteristics and capabilities before implementing them to promote the most efficient urine sediment testing.

A major limitation of the present study is that the screening performance of the UF‐1500 for clinical diagnosis of urinary tract infections and glomerulonephritis was not investigated. In addition, specific research parameters such as RBC‐Info, UTI‐Info, and BACT‐Info can be displayed on both the UF‐1500, as well as the UF‐5000. The research parameters have been validated to be clinically useful on the UF‐5000,^7^, ^8^, ^10^ and will need to be evaluated on the UF‐1500 in the future. However, in this study, we observed that the concordance rates between the UF‐1500 and manual microscopy were comparable to those of the UF‐5000. This suggests that the UF‐1500 can also be an effective screening tool for various renal clinical conditions.

## 5 CONCLUSIONS

In conclusion, the performance of the UF‐1500 is comparable to that of the well‐ established UF‐5000 analyzer and it demonstrates sufficient capability to be used in clinical practice.

## AUTHOR CONTRIBUTIONS

Yoshifumi Morita were principal investigators, designed the study, wrote the draft, and obtained funding. Yoshifumi Morita, Rin Yokoyama, Masami Tanaka, Naru Nakatsuka, and Takashi Hisasue had roles in data collection. Yoshikazu Ono and Makoto Kurano supervised the study and revised the article. All authors agreed to be accountable for all aspects of work.

## FUNDING INFORMATION

This study was funded by Sysmex Corporation.

## CONFLICT OF INTEREST STATEMENT

The present study was performed under a collaborative research project at the University of Tokyo Hospital with Sysmex Corporation.

## ETHICS STATEMENT

The current study was performed under the ethical guidelines outlined in the Declaration of Helsinki. The University of Tokyo Medical Research Center Ethics Committee (2019300NI‐6) approved the study design.

## PATIENT CONSENT STATEMENT

Informed consent was obtained in the form of an opt‐out option on a website as follows; Patients were informed about the study on the website, and those who did not wish to be enrolled in the study were excluded.

## PERMISSION TO REPRODUCE MATERIAL FROM OTHER SOURCES

None.

## CLINICAL TRIAL REGISTRATION

None.

## Supporting information


Figure S1.

## Data Availability

Data are available upon reasonable request.
