# Supplementary material for: Evaluation of the fully automated urine particle analyzer UF‐1500
Source: J Clin Lab Anal. 2023 Dec 2;37(23-24):e24993. doi: 10.1002/jcla.24993 (PMC10756941; doi:10.1002/jcla.24993)
Supplement: Supplementary file 1 — Figure S1. [file JCLA-37-e24993-s001.pdf]

Figure S1

A

RBC

UF-5000 (/HPF) \ manual microscopy (/HPF)	<1	1-4	5-9	10-19	20-29	30-49	50-99	≥100
≥100	0	0	0	0	0	0	0	60
50-99	1	0	0	0	0	1	19	6
30-49	0	1	0	1	3	21	8	0
20-29	2	1	1	0	10	4	2	0
10-19	8	4	5	27	11	4	0	0
5-9	11	9	18	21	1	0	0	0
1-4	53	90	14	3	0	0	0	0
<1	209	18	1	0	0	0	0	0

B

WBC

UF-5000 (/HPF) \ manual microscopy (/HPF)	<1	1-4	5-9	10-19	20-29	30-49	50-99	≥100
≥100	0	0	0	0	0	0	3	49
50-99	0	0	1	0	0	2	37	3
30-49	0	0	0	1	2	18	9	1
20-29	0	0	1	8	21	6	2	0
10-19	1	1	7	29	11	7	0	0
5-9	0	8	32	13	3	0	0	0
1-4	34	100	17	2	1	0	0	0
<1	199	19	0	0	0	0	0	0

C

EC

UF-5000 (/HPF) \ manual microscopy (/HPF)	<1	1-4	5-9	10-19	20-29	30-49	50-99	≥100
≥100	0	0	0	0	0	0	0	0
50-99	0	0	0	0	0	0	0	0
30-49	0	0	0	0	1	2	0	0
20-29	0	0	0	2	3	4	0	0
10-19	1	0	6	10	4	4	0	0
5-9	1	5	18	20	4	1	0	0
1-4	106	83	29	1	1	0	0	0
<1	324	17	1	0	0	0	0	0

D

Squa. EC

UF-5000 (/HPF) \ manual microscopy (/HPF)	<1	1-4	5-9	10-19	20-29	30-49	50-99	≥100
≥100	0	0	0	0	0	0	0	0
50-99	0	0	0	0	0	0	0	0
30-49	0	0	0	0	1	2	0	0
20-29	0	0	0	1	3	4	0	0
10-19	1	0	4	8	2	4	0	0
5-9	1	0	13	19	3	1	0	0
1-4	26	48	18	3	3	0	0	0
<1	453	24	5	1	0	0	0	0

E

CAST

UF-5000 (/LPF) \ manual microscopy (/LPF)	<1	1-4	5-9	10-19	20-29	30-49	50-99	≥100
≥100	0	0	0	0	0	0	0	0
50-99	0	0	1	0	0	1	0	0
30-49	0	0	0	0	0	0	0	0
20-29	1	0	2	0	0	0	0	0
10-19	1	0	2	2	0	0	0	0
5-9	9	2	9	2	0	1	0	0
1-4	69	10	15	2	0	0	0	0
<1	252	1	1	0	0	0	0	0

F

BACT

UF-5000 (/μL) \ manual microscopy	-	1+	2+	3+
≥10000	0	3	29	37
1000-9999	4	47	15	3
100-999	65	64	0	1
<100	375	4	0	1

Supplemental Figure S1. Comparison results of quantitative parameters in UF-5000.
Consistency between the UF-5000 and manual microscopy in RBC (A), WBC (B), EC (C), Squa.EC (D), CAST (E), and BACT (F) is shown in the box correlation diagrams. The red line represents the cut-off value.

Red blood cell, RBC; white blood cell, WBC; epithelial cell, EC; squamous epithelial cell, Squa.EC; bacteria, BACT

Figure S2

A

WBC Clumps

UF-5000 (/HPF)	-	+
≥1	91	17
<1	534	6

manual microscopy

B

Non SEC

UF-5000 (/HPF)	<1	1-4	5-9	10-19	20-29	30-49	50-99	≥100
≥100	0	0	0	0	0	0	0	0
50-99	0	0	0	0	0	0	0	0
30-49	0	0	0	0	0	0	0	0
20-29	0	0	0	0	0	0	0	0
10-19	0	0	0	0	0	0	0	0
5-9	2	1	3	1	0	0	0	0
1-4	107	20	10	1	0	0	0	0
<1	487	14	1	1	0	0	0	0

manual microscopy (/HPF)

C

Hy.CAST

UF-5000 (/LPF)	<1	1-4	5-9	10-19	20-29	30-49	50-99	≥100
≥100	0	0	0	0	0	0	0	0
50-99	0	0	0	0	0	0	0	0
30-49	0	1	0	0	0	1	0	0
20-29	0	0	1	0	0	0	0	0
10-19	0	1	1	1	0	0	0	0
5-9	4	0	1	2	0	1	0	0
1-4	53	15	15	2	0	0	0	0
<1	279	6	1	1	0	0	0	0

manual microscopy (/LPF)

D

Path.CAST

UF-5000 (/LPF)	<1	1-4	5-9	10-19	20-29	30-49	50-99	≥100
≥100	0	0	0	0	0	0	0	0
50-99	0	0	0	0	0	0	0	0
30-49	0	1	0	0	0	0	0	0
20-29	1	0	0	0	0	0	0	0
10-19	2	1	0	0	0	0	0	0
5-9	5	1	2	0	0	0	0	0
1-4	45	5	0	0	0	0	0	0
<1	483	3	1	0	0	0	0	0

manual microscopy (/LPF)

E

X'TAL

UF-5000 (/HPF)	-	1+	2+	3+
≥10	0	10	5	3
5-9	2	5	2	0
1-4	11	5	0	0
<1	592	12	1	0

manual microscopy

F

YLC

UF-5000 (/HPF)	-	1+	2+	3+
≥100	10	0	1	0
10-99	56	4	0	0
1-9	33	1	0	0
<1	543	0	0	0

manual microscopy

G

SPERM

UF-5000 (/HPF)	-	1+	2+	3+
≥100	0	0	1	0
10-99	0	3	0	0
1-9	1	2	0	0
<1	636	5	0	0

manual microscopy

H

MUCUS

UF-5000 (/LPF)	-	+
≥1	48	83
<1	428	89

manual microscopy

Supplemental Figure S2. Comparison results of semi-quantitative or qualitative parameters in UF-5000. Consistency between the UF-1500 and manual microscopy in WBC clumps (A), Non SEC (B), Hy.CAST (C), Path.CAST (D), X'TAL (E), YLC (F), SPERM (G), and MUCUS (H) is shown in the box correlation diagrams. The red line represents the cut-off value.

Non-squamous epithelial cell, Non SEC; hyaline cast, Hy.CAST; non-hyaline cast, Path.CAST; crystal, X'TAL; yeast-like-cell, YLC; spermatozoa, SPERM
